# Epigenome‐wide association study of sarcopenia: findings from the Hertfordshire Sarcopenia Study (HSS)

**DOI:** 10.1002/jcsm.12876

**Published:** 2021-12-04

**Authors:** Elie Antoun, Emma S. Garratt, Andrea Taddei, Mark A. Burton, Sheila J. Barton, Phil Titcombe, Leo D. Westbury, Alicia Baczynska, Eugenia Migliavacca, Jerome N. Feige, Holly E. Sydall, Elaine Dennison, Richard Dodds, Helen C. Roberts, Peter Richardson, Avan A. Sayer, Sarah Shaw, Cyrus Cooper, Joanna D. Holbrook, Harnish P. Patel, Keith M. Godfrey, Karen A. Lillycrop, the EpiGen Global Research Consortium

**Affiliations:** ^1^ Human Development and Health Academic Unit, Faculty of Medicine University of Southampton Southampton UK; ^2^ Biological Sciences University of Southampton Southampton UK; ^3^ NIHR Southampton Biomedical Research Centre University of Southampton & University Hospital Southampton NHS Foundation Trust Southampton UK; ^4^ Benevolent AI London UK; ^5^ MRC Lifecourse Epidemiology Centre University of Southampton Southampton UK; ^6^ Academic Geriatric Medicine, Faculty of Medicine University of Southampton Southampton UK; ^7^ Nestle Research, EPFL Innovation Park Lausanne Switzerland; ^8^ AGE Research Group, Translational and Clinical Research Institute, Faculty of Medical Sciences Newcastle University Newcastle upon Tyne UK; ^9^ NIHR Newcastle Biomedical Research Centre Newcastle University and Newcastle upon Tyne Hospitals NHS Foundation Trust Newcastle upon Tyne UK

**Keywords:** DNA methylation, Sarcopenia, EZH2, Myoblasts

## Abstract

**Background:**

Sarcopenia is the age‐related loss of muscle mass, strength, and function. Epigenetic processes such as DNA methylation, which integrate both genetic and environmental exposures, have been suggested to contribute to the development of sarcopenia. This study aimed to determine whether differences in the muscle methylome are associated with sarcopenia and its component measures: grip strength, appendicular lean mass index (ALMi), and gait speed.

**Methods:**

Using the Infinium Human MethylationEPIC BeadChip, we measured DNA methylation in *vastus lateralis* muscle biopsies of 83 male participants (12 with sarcopenia) with a mean (standard deviation) age of 75.7 (3.6) years from the Hertfordshire Sarcopenia Study (HSS) and Hertfordshire Sarcopenia Study extension (HSSe) and examined associations with sarcopenia and its components. Pathway, histone mark, and transcription factor enrichment of the differentially methylated CpGs (dmCpGs) were determined, and sodium bisulfite pyrosequencing was used to validate the sarcopenia‐associated dmCpGs. Human primary myoblasts (*n* = 6) isolated from *vastus lateralis* muscle biopsies from male individuals from HSSe were treated with the EZH2 inhibitor GSK343 to assess how perturbations in epigenetic processes may impact myoblast differentiation and fusion, measured by PAX7 and MYHC immunocytochemistry, and mitochondrial bioenergetics determined using the Seahorse XF96.

**Results:**

Sarcopenia was associated with differential methylation at 176 dmCpGs (false discovery rate ≤ 0.05) and 141 differentially methylated regions (Stouffer ≤ 0.05). The sarcopenia‐associated dmCpGs were enriched in genes associated with myotube fusion (*P* = 1.40E‐03), oxidative phosphorylation (*P* = 2.78E‐02), and voltage‐gated calcium channels (*P* = 1.59E‐04). ALMi was associated with 71 dmCpGs, grip strength with 49 dmCpGs, and gait speed with 23 dmCpGs (false discovery rate ≤ 0.05). There was significant overlap between the dmCpGs associated with sarcopenia and ALMi (*P* = 3.4E‐35), sarcopenia and gait speed (*P* = 4.78E‐03), and sarcopenia and grip strength (*P* = 7.55E‐06). There was also an over‐representation of the sarcopenia, ALMi, grip strength, and gait speed‐associated dmCpGs with sites of H3K27 trimethylation (all *P* ≤ 0.05) and amongst EZH2 target genes (all *P* ≤ 0.05). Furthermore, treatment of human primary myoblasts with the EZH2 inhibitor GSK343 inhibitor led to an increase in PAX7 expression (*P* ≤ 0.05), decreased myotube fusion (*P* = 0.043), and an increase in ATP production (*P* = 0.008), with alterations in the DNA methylation of genes involved in oxidative phosphorylation and myogenesis.

**Conclusions:**

These findings show that differences in the muscle methylome are associated with sarcopenia and individual measures of muscle mass, strength, and function in older individuals. This suggests that changes in the epigenetic regulation of genes may contribute to impaired muscle function in later life.

## Introduction

Sarcopenia, defined as the loss of muscle mass, strength, and function with advancing age, is associated with a number of adverse physical and metabolic changes that contribute to morbidity, impaired quality of life, increased health care costs, and mortality. Although several operational definitions of sarcopenia are used worldwide,^1^, ^2^ there is general consensus that defining sarcopenia relies on low muscle function (either weak muscle strength or impaired physical performance, i.e. slower gait speed) in combination with low whole‐body or appendicular lean mass.^1^, ^3^, ^4^, ^5^ Prevalence estimates have been reported to be between 1–29% amongst community‐dwelling older adults, 14–33% for those in long‐term care, and 10% for those in acute hospital.^3^, ^6^

A decline in muscle mass, strength, and function is a fundamental consequence of ageing; however, there is significant variability between individuals in the rate of loss in old age. Some of the variability can be explained by fixed genetic factors,^7^, ^8^ but much of the remaining variation is unexplained. There is growing evidence that suggests epigenetic processes play a prominent role in the development of many complex diseases.^9^ Processes such as DNA methylation induce heritable changes in gene expression without a change in nucleotide sequence.^10^ As inter‐individual DNA methylation is specified by the interaction of both genotypic and environmental influences,^11^ changes in DNA methylation within skeletal muscle may provide novel insights into the variability in muscle ageing, as well as provide powerful biomarkers when compared with either genotype or lifestyle factors alone. To date, a number of human studies have compared DNA methylation in muscle tissue from young versus old individuals^12^, ^13^, ^14^, ^15^, ^16^ and reported differential methylation of genes involved in axon guidance,^12^, ^13^ cytoskeletal function,^13^, ^16^ cell adhesion,^12^, ^13^ muscle contraction,^15^ calcium signalling,^15^ and mTOR signalling.^12^ DNA methylation in muscle tissue in individuals with sarcopenia compared with healthy aged‐matched controls has not previously been reported. A comparison of the muscle transcriptome in 119 older men with sarcopenia versus age‐matched controls from Singapore, Hertfordshire UK, and Jamaica demonstrated that the major transcriptional signature of sarcopenia was mitochondrial bioenergetic dysfunction in skeletal muscle, with down‐regulation of oxidative phosphorylation genes.^17^ However, whether these transcriptional changes are mediated through changes in DNA methylation is not known. Here, we sought to identify DNA methylation changes in muscle associated with sarcopenia, and its components, namely, grip strength, appendicular lean mass index (ALMi), and gait speed.

## Methods

### Study design

All participants were recruited from the Hertfordshire Cohort Study (HCS),^18^ a retrospective cohort study based in the UK designed to investigate life course influences on muscle function in community‐dwelling older people. DNA was analysed from 40 male participants from the first phase of the study, the Hertfordshire Sarcopenia Study (HSS),^19^, ^20^ and from the male participants of the second extension phase of the study (*n* = 43), herein termed HSSe.^21^ The 40 males from the HSS and the 43 males from the HSSe were the only samples with sufficient DNA for both genome‐wide methylation analysis and pyrosequencing. All participants gave written informed consent, and the study was approved by the Hertfordshire Research Ethics Committee (07/Q0204/68). Sarcopenia was defined according to the European Working Group on Sarcopenia in Older People (EWGSOP)^3^ criteria, with the following thresholds: ALMi (ALM/height^2^) ≤ 7.23 kg/m^2^ for men and ≤5.67 kg/m^2^ for women; grip strength < 30 kg for men and <20 kg for women; and walking speed ≤ 0.8 m/s. Participants were classed as healthy controls (normal ALMi, gait speed, and grip strength) or as having sarcopenia (low ALMi and low gait speed and/or low grip strength).

### Procedures

Body composition (appendicular lean mass) was assessed by dual‐energy X‐ray absorptiometry (DXA) (Hologic Discovery, software version 12.5). Isometric grip strength (kilograms) was measured three times in each hand using a Jamar handheld hydraulic dynamometer (Promedics, UK), and the highest value of six measures used.^22^ Customary walking speed was measured over a 3 m course. Percutaneous muscle biopsies of the *vastus lateralis* were conducted after an overnight fast under local anaesthetic using a Weil–Blakesley conchotome.^23^

### Infinium Human MethylationEPIC BeadChip array

Genomic DNA was extracted from muscle from HSS participants using the QIAamp DNA mini kit (Qiagen) and from HSSe participants using the high salt method.^24^ A total of 750 ng of genomic DNA was treated with sodium bisulfite using Zymo EZ DNA Methylation‐Gold kit (ZymoResearch, USA) and hybridized to the Infinium Human MethylationEPIC BeadChip array (Illumina, Inc., USA) at the Centre for Molecular Medicine and Therapeutics (http://www.cmmt.ubc.ca).

### Infinium Human MethylationEPIC BeadChip array data processing

EPIC array data were processed using the Bioconductor package minfi^25^ in R (version 3.4.2). See details described in Supporting Information, *Data* S1. After pre‐processing and QC, 77 samples for the muscle tissue analysis remained, and 10 for the myoblast analysis.

### Infinium Human MethylationEPIC BeadChip array data analysis

Robust regression models were run using limma (v3.38.3).^26^ Models were adjusted for age, and surrogate variables, to account differences in cellular heterogeneity. The analysis was controlled for multiple testing with the Benjamini–Hochberg adjustment for false discovery rate (FDR), using an FDR < 0.05. See full details in *Data* S1.

### Gene ontology, histone, and transcription factor enrichment analysis

Protein–protein interaction (PPI) networks were carried out using the Search Tool for the Retrieval of Interacting Genes/Proteins (STRING) and visualized in Cytoscape. Large networks were segmented using the MCODE algorithm,^27^ and gene ontology (GO) enrichment determined using BiNGO.^28^ Enrichment of differentially methylated CpGs (dmCpGs) amongst regions of histone modifications and transcription factor binding sites was assessed using the ChIP‐seq peak data from ENCODE in male human skeletal muscle tissue (https://www.encodeproject.org).

### Muscle epigenetic age estimator

Epigenetic age acceleration was calculated as the residuals of regressing the epigenetic age estimated by the muscle epigenetic age estimator (MEAT)^14^ over chronological age.

### RNA sequencing and analysis

RNA was extracted from frozen muscle tissue using the mirVana™ miRNA Isolation Kit (Ambion, Life Technologies), and RNA sequencing carried out as described in Migliavacca *et al*.^17^

### Pyrosequencing methylation analysis

Quantitative DNA methylation analysis was carried out by pyrosequencing, described in *Data* S1. Primer sequences are shown in Supporting Information, *Table* S1.

### Isolation of myoblast cells from muscle biopsies

Details of myoblast isolation and culture are described in *Data* S1. Briefly, biopsies were minced (three with sarcopenia and three healthy aged‐matched controls), digested in 0.5 mg/mL collagenase (Sigma), before pre‐plating to remove fibroblasts, and sorting using CD56 MicroBeads (Miltenyi Biotech).^29^ Isolation of myogenic cells was confirmed by immunocytochemistry, which showed ≥96% of cells as CD56 positive after sorting. Cells were grown in either proliferation medium [Dulbecco's modified Eagle's medium (DMEM) containing 20% foetal bovine serum (FBS), 10% horse serum (HS), 1% chick embryo extract, and 1% penicillin/streptomycin (P/S)] or differentiation media (DMEM containing 2% HS and 1% P/S). Human primary myoblasts were treated with GSK343 (MedChemExpress), an inhibitor of EZH2,^30^ at 20 nM, 200 nM, and 2 μM after the initiation of differentiation over a 10 day period.

### Immunocytochemistry

Cells were stained for myosin heavy chain (MYHC), PAX7, and 4′,6‐diamidino‐2‐phenylindole (DAPI) (1 μg/mL). See details in *Data* S1. To calculate the fusion index, the number of nuclei within myotubes (containing 2+ nuclei) was counted and the ratio of this number to the total number of nuclei was determined.

### Metabolic flux assay

Mitochondrial bioenergetics were measured using the Agilent Seahorse XF96 Mito Stress Test (details in *Data* S1). After measurement, cells were lysed and a protein assay was carried out for normalization. Each data point represents the mean ± standard deviation of six replicates for each condition.

### Statistical analysis

All statistical analyses were carried out in R (version 3.4.2). Demographic characteristics were compared between controls and those with sarcopenia using the Mann–Whitney *U* tests. The hypergeometric distribution probability test was used to test the significance of the overlap between dmCpGs associated with different measures of muscle mass/function. Fisher's exact test was used to test the enrichment of dmCpGs amongst the different histone modifications, chromatin enhancer states, and genomic regions relative to CpG islands. Linear models were fitted to the pyrosequencing data including age as a covariate. Correlation analysis of the methylation and gene expression data was carried out using Spearman's correlation. Statistical analysis of the GSK343‐treated cell cultures was carried out using the Wilcoxon signed‐rank test for the immunocytochemistry and paired *t*‐test for the metabolic flux assays, with data from sarcopenia and control myoblasts analysed as one group to maximize statistical power.

## Results

### Participant characteristics

Of the 83 men who had muscle tissue available, 77 samples passed downstream quality control and were included in further analysis. Participant characteristics are given in *Table* 1. Men who had sarcopenia (*n* = 11) were older (*P* = 0.019) and had a lower body mass index (*P* < 0.001) compared with those who did not have sarcopenia. As expected, men with sarcopenia also had lower measures of muscle mass [total lean mass, appendicular lean mass (ALM), and ALMi; all *P* < 0.001] and reduced muscle function [gait speed (*P* < 0.001)] and grip strength (*P* = 0.001) compared with those who did not have sarcopenia.

**Table 1:** Participant characteristics

	Control (*n* = 41)	Sarcopenia (*n* = 11)	Total (*n* = 77^a^)	
Age (years)	74.49 ± 3.25	77.12 ± 3.74	75.66 ± 3.55	* *P* = 1.90 × 10^−2^
Height (cm)	172.32 ± 5.77	171.68 ± 9.81	172.22 ± 6.12	*P* = 9.22 × 10^−1^
Weight (kg)	83.45 ± 12.81	76.06 ± 12.14	79.10 ± 12.32	*** *P* = 1.00 × 10^−3^
BMI (kg/m ^2^)	28.04 ± 3.66	25.74 ± 2.86	26.10 ± 3.47	**** *P* = 1.28 × 10^−4^
Total lean mass (kg)	55.33 ± 6.94	47.82 ± 5.45	51.58 ± 7.29	**** *P* = 2.96 × 10^−7^
ALM (kg)	24.42 ± 2.98	20.03 ± 2.68	22.35 ± 3.46	**** *P* = 4.06 × 10^−10^
Fat mass (kg)	24.15 ± 8.02	24.61 ± 9.05	23.77 ± 7.81	*P* = 5.64 × 10^−1^
Grip strength (kg)	38.05 ± 6.86	29.00 ± 8.72	36.19 ± 7.15	*** *P* = 1.00 × 10^−3^
Gait speed (m/s)	1.08 ± 018	0.82 ± 0.26	1.05 ± 021	**** *P* = 3.61 × 10^−4^
ALMi (kg/m^2^)	8.21 ± 0.76	6.77 ± 0.36	7.53 ± 0.98	**** *P* = 5.92 × 10^−15^

ALM, appendicular lean mass; ALMi, appendicular lean mass index; BMI, body mass index.

^a^ Twenty participants were not classed as healthy controls or as having sarcopenia using the European Working Group on Sarcopenia in Older People definition; however, they were included in the continuous analyses of ALMi, grip strength, and gait speed.

* *P*‐value ≤ 0.05.

** *P*‐value ≤ 0.01.

*** *P*‐value ≤ 0.001.

**** *P*‐value ≤ 0.0001.

### Identification of differentially methylated CpGs in muscle biopsies associated with sarcopenia

**Figure 1:**
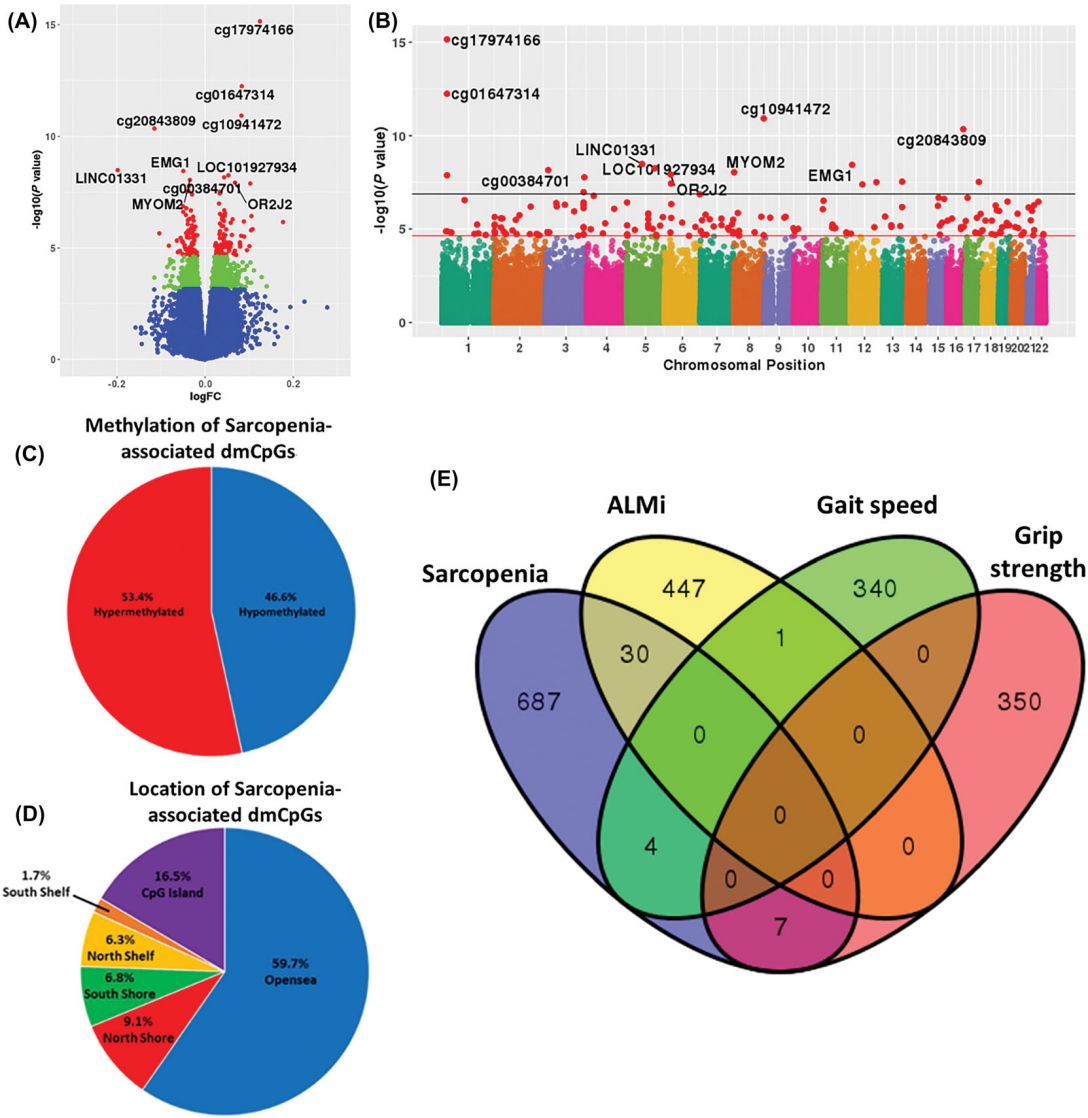
*(A)* Volcano plot of the differential methylation analysis with respect to sarcopenia, with the significant probes (FDR < 0.05) highlighted in red and probes with an FDR < 0.25 highlighted in green. The top 10 dmCpGs have been annotated. *(B)* Manhattan plot for the differential methylation analysis with respect to sarcopenia. All significant sarcopenia‐associated dmCpGs are shown as bold red points, with the top 10 dmCpGs annotated. Black line: Bonferroni threshold (*P*‐value = 1.33 × 10^−7^); red line: FDR threshold (*P*‐value = 2.34 × 10^−5^). *(C)* Pie chart showing the proportions of dmCpGs showing increased or decreased methylation associated with sarcopenia. *(D)* Pie chart showing the proportions of the locations of the sarcopenia‐associated dmCpGs. *(E)* Venn diagram indicating the overlap in the number of CpGs (FDR < 0.2) associated with sarcopenia and the multiple measures of muscle mass.

There were significant (FDR, *P* < 0.05) associations between DNA methylation and sarcopenia, with 176 CpGs significantly associated with sarcopenia (*Tables* 2 and S2). The top two dmCpGs were located in an intergenic region on chromosome 1 (cg17974166, FDR = 2.67 × 10^−10^, and cg01647314, FDR = 1.06 × 10^−7^) (*Figure* 1A + 1B); 53.4% of the dmCpGs showed hypermethylation in those with sarcopenia (*Figure* 1C), with a significant over‐representation of dmCpGs in the OpenSea (*P* = 0.0188) and CpG island (*P* = 1.28 × 10^−5^) regions (*Figure* 1D).

**Table 2:** Top 25 sarcopenia‐associated differentially methylated CpGs

Probe	logFC	Average methylation	FDR	Gene
cg17974166	0.1244	0.1246	2.67 × 10^−10^	
cg01647314	0.0831	0.1640	1.06 × 10^−7^	
cg10941472	0.0823	0.1102	1.49 × 10^−6^	
cg20843809	−0.1151	0.8705	4.22 × 10^−6^	
cg07236061	−0.1987	0.8646	2.28 × 10^−4^	LINC01331
cg22843429	−0.0497	0.8661	2.28 × 10^−4^	EMG1
cg03622584	0.0528	0.6245	3.03 × 10^−4^	LOC101927934
cg00384701	0.0430	0.3116	3.28 × 10^−4^	
cg10977501	−0.0346	0.9088	3.81 × 10^−4^	MYOM2
cg27191906	0.0676	0.6362	4.51 × 10^−4^	OR2J2
cg00172812	0.1024	0.2311	4.51 × 10^−4^	
cg00102685	−0.0368	0.9359	5.33 × 10^−4^	
cg08344114	−0.0378	0.5612	7.77 × 10^−4^	
cg27473406	−0.0416	0.3952	7.77 × 10^−4^	
cg26945376	0.0331	0.1893	7.77 × 10^−4^	CLIP1
cg10956589	0.0330	0.4653	8.66 × 10^−4^	
cg03532253	−0.0296	0.8865	9.09 × 10^−4^	SUOX
cg25441771	0.0316	0.0468	2.20 × 10^−3^	MCCC1
cg16307778	−0.0501	0.8696	2.75 × 10^−3^	
cg15744876	−0.0428	0.5705	3.17 × 10^−3^	
cg04344695	0.0434	0.4816	3.66 × 10^−3^	CA12
cg27253454	−0.0323	0.7946	3.66 × 10^−3^	USP43
cg14659184	−0.0499	0.8841	4.01 × 10^−3^	LINC00925
cg20266770	0.0451	0.2541	4.45 × 10^−3^	
cg25963061	0.0336	0.7137	4.59 × 10^−3^	UBQLNL

FC, fold change; FDR, false discovery rate.

To understand the contribution of muscle mass, strength, and function to the methylation signature associated with sarcopenia, we analysed DNA methylation with respect to ALMi, grip strength, and gait speed as continuous variables. ALMi was associated with 71 dmCpGs; grip strength with 49 dmCpGs; and gait speed with 23 dmCpGs (*Tables* S3–S5). The top dmCpG associated with ALMi was cg22350027, located in the body of the phospholipase C‐like protein 2 (*PLCL2*) gene (FDR = 9.68 × 10^−6^), while the top dmCpGs associated with grip strength and gait speed were located in the intergenic region of chromosome 17 (FDR = 0.0003) and chromosome 1 (FDR = 0.0017), respectively. There was significant overlap between the dmCpGs associated with sarcopenia and ALMi (*P* = 3.40 × 10^−35^), sarcopenia and gait speed (*P* = 4.78 × 10^−3^), and sarcopenia and grip strength (*P* = 7.55 × 10^−6^) (*Table* S6, *Figure* 1E). However, there was no overlap between the dmCpGs associated with ALMi, grip strength, or gait speed.

### Sarcopenia is not associated with accelerated epigenetic age

To determine whether the methylation changes associated with sarcopenia represented accelerated muscle ageing, epigenetic age acceleration was calculated.^14^ Epigenetic age as determined by MEAT was strongly correlated with chronological age (Supporting Information, *Figure* S2; Pearson's *r* = 0.421, *P* = 0.0001). However, there were no significant associations of accelerated epigenetic ageing with sarcopenia status, ALMi, gait speed, or grip strength (*Table* S7).

### Sarcopenia and the individual measures of muscle mass, strength, and function are associated with multiple differentially methylated regions

Regional analysis identified differentially methylated regions (DMRs) associated with sarcopenia, ALMI, grip strength, and gait speed (*Tables* S8–S11). Sarcopenia was associated with 141 DMRs (Stouffer < 0.05), with the top DMR located within the promoter region of the Methylcrotonyl‐CoA Carboxylase 1 (*MCCC1*) gene, consisting of 13 CpGs (Stouffer = 3.62 × 10^−12^). ALMi was associated with 135 DMRs, with the top DMR located within the zinc finger protein 57 (*ZFP57*) gene. Grip strength was associated with 28 DMRs, with the top DMR located within the claudin 10 (*CLDN10*) gene (Stouffer = 8.29 × 10^−4^). Gait speed was associated with 24 DMRs, with the top DMR located in an intergenic region on chromosome 1 (Stouffer = 7.53 × 10^−4^). There were three DMRs associated with both sarcopenia and ALMi, but no DMRs that overlapped between sarcopenia and grip strength, or sarcopenia and gait speed.

### Correlation between DNA methylation and gene expression in skeletal muscle tissue

Of the 77 samples analysed by EPIC DNA methylation arrays, 34 muscle samples had previously been analysed using total RNAseq.^17^ Therefore, to explore the correlation between DNA methylation and gene expression, we examined the sarcopenia‐associated dmCpGs with an FDR < 0.2 annotated to a gene (*n* = 470), with the RNAseq data, removing transcripts with low expression across all samples. This resulted in 298 transcript–CpG pairs. Correlation analysis of the transcript–CpG pairs revealed 29 with a significant correlation between methylation and expression at an FDR < 0.2 (*Table* 3). There were multiple dmCpGs within *MCCC1*, which were associated with transcript levels: three CpGs in the body of the *MCCC1* gene (cg08395365, cg22211233, and cg00161968) and one within 200 bp of the *MCCC1* TSS (cg00890010) were negatively associated with *MCCC1* expression, while cg23476885, located in the body of the *MCCC1* gene, was positively associated with *MCCC1* expression.

**Table 3:** Correlation between DNA methylation and RNA expression (false discovery rate < 0.2)

Probe	Spearman's *ρ*	*P*‐value	FDR	Gene	CpG position
**dmCpGs**					
cg20108671	−0.6327	8.57E‐05	0.0260	SCAPER	5′UTR
cg03854273	−0.5939	2.80E‐04	0.0339	LPCAT3	Body
cg13403462	−0.5875	3.36E‐04	0.0339	NECAB3	TSS200
cg24859375	−0.5438	1.06E‐03	0.0786	LPCAT3	1st exon
cg04180086	0.5355	1.30E‐03	0.0786	IRX4	Body
cg01554606	−0.5150	2.09E‐03	0.0981	MYLK	Body
cg08395365	−0.5001	2.92E‐03	0.0981	MCCC1	Body
cg07655627	−0.4967	3.14E‐03	0.0981	NUDT12	Body
cg09166085	−0.4937	3.36E‐03	0.0981	NUDT12	3′UTR
cg02976617	−0.4934	3.38E‐03	0.0981	NUDT12	Body
cg22211233	−0.4909	3.56E‐03	0.0981	MCCC1	Body
cg23423191	−0.4860	3.95E‐03	0.0997	STK10	5′UTR
cg00617927	0.4756	4.90E‐03	0.1073	COL27A1	TSS1500
cg03073264	−0.4750	4.96E‐03	0.1073	NUDT12	Body
cg08767025	0.4652	6.03E‐03	0.1219	SOX5	TSS200
cg22350027	0.4597	6.72E‐03	0.1273	PLCL2	Body
cg16938504	−0.4564	7.18E‐03	0.1279	RAB40B	TSS1500
cg00890010	−0.4503	8.07E‐03	0.1285	MCCC1	TSS200
cg13665998	−0.4484	8.36E‐03	0.1285	NUDT12	3′UTR
cg07666882	−0.4454	8.85E‐03	0.1285	NUDT12	TSS1500
cg18172877	0.4451	8.91E‐03	0.1285	IRX4	5′UTR
cg06105925	−0.4420	9.43E‐03	0.1299	NUDT12	TSS1500
cg09414187	0.4353	1.07E‐02	0.1406	CENPV	Body
cg20279471	−0.4292	1.19E‐02	0.1506	NUDT12	Body
cg00161968	−0.4264	1.25E‐02	0.1519	MCCC1	Body
cg23476885	0.4191	1.43E‐02	0.1663	MCCC1	Body
cg22855016	−0.4108	1.65E‐02	0.1847	NFYC	Body
cg15020502	−0.4072	1.75E‐02	0.1888	ETS2	TSS1500
cg00575744	−0.4053	1.81E‐02	0.1888	HLA‐DMB	1st exon
**DMRs**					
cg11689786	−0.5251	1.66E‐03	0.1623	MYL6B	5′UTR
cg07655627	−0.4967	3.14E‐03	0.1623	NUDT12	TSS1500
cg15206834	−0.4949	3.27E‐03	0.1623	NUDT12	TSS1500
cg09166085	−0.4937	3.36E‐03	0.1623	NUDT12	TSS200
cg02976617	−0.4934	3.38E‐03	0.1623	NUDT12	5′UTR
cg03073264	−0.4750	4.96E‐03	0.1983	NUDT12	5′UTR

dmCpGs, differentially methylated CpGs; DMRs, differentially methylated regions; FDR, false discovery rate.

Correlation between the CpGs within the DMRs also showed low congruence between DNA methylation and gene expression. Of the 279 CpGs, located within the DMRs, related to a gene with a normalized readcount > 20 in at least 5 samples, 6 CpGs correlated with gene expression at an FDR < 0.2 (*Table* 3).

### Validation

**Figure 2:**
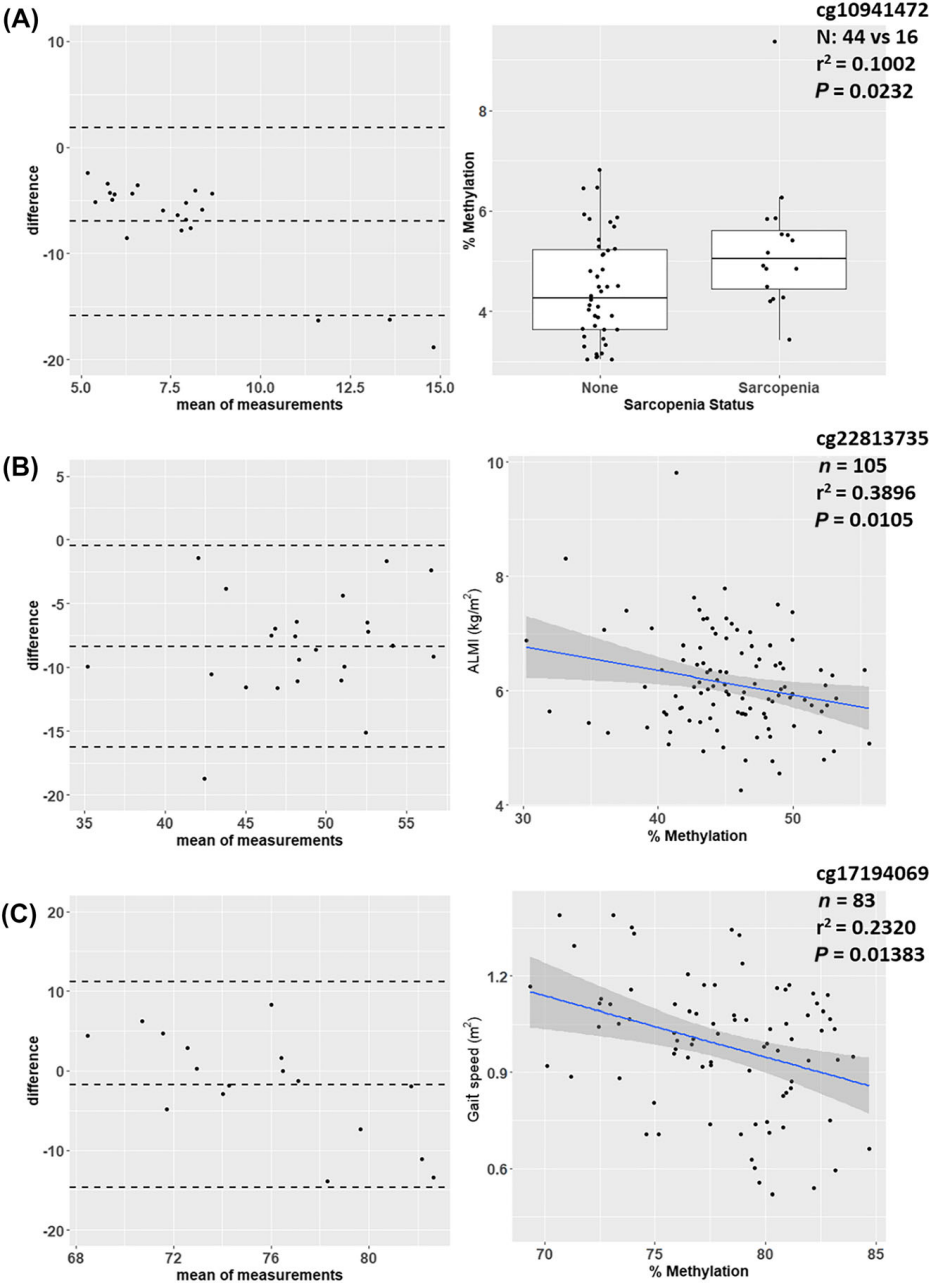
Bisulfite pyrosequencing validation of top hits associated with *(A)* sarcopenia, *(B)* ALMi, and *(C)* gait speed. Bland–Altman plots (left‐hand panels) show the differences between the two technologies (pyrosequencing and array), with the majority of the points within the limits of agreement. All three CpGs show significant associations with *(A)* sarcopenia, *(B)* ALMi, and *(C)* gait speed, in the same direction of change as seen on the array.

Sodium bisulfite pyrosequencing was performed to validate cg10941472 (*Figure* 2A), cg22813735 (*Figure* 2B), and cg17194069 (*Figure* 2C), top hits associated with sarcopenia, ALMi, and gait speed, respectively, whose genomic context allowed sequencing primers to be designed. Bland–Altman plots comparing the methylation values between the pyrosequencer and the array for these CpG sites showed that the majority of the data points from the two methods lay within the limits of agreement. Moreover, analysis of the associations between the methylation status of the three CpGs, measured by pyrosequencing, and phenotypic parameters also replicated the array analysis, with cg10941472 significantly associated with sarcopenia status (*P* = 0.0232), cg22813735 negatively associated with ALMi (*P* = 0.0105), and cg17194069 negatively associated with gait speed (*P* = 0.01383).

### Sarcopenia‐associated differentially methylated CpGs were enriched in pathways associated with cell adhesion, energy production, and voltage‐gated calcium channels

To gain an understanding of the functional significance of the methylation changes, genes associated with the dmCpGs of FDR < 0.2 were inputted into STRING to generate a PPI network (*Figure* S3a). The PPI enrichment *P*‐value for the network was 0.0121, indicating biological connection between the proteins. Ten significant GO terms were over‐represented in the PPI network (FDR < 0.05; *Table* S12), with the top term being homophilic cell adhesion (FDR = 0.0014), a process essential for myotube fusion. Subdivision of the network using MCODE identified clusters enriched for GO terms including phosphorus metabolic process (FDR = 1.20 × 10^−3^), oxidative phosphorylation (FDR = 2.78 × 10^−2^), and voltage‐gated Ca^2+^ channel activity (FDR = 1.59 × 10^−4^) (*Table* S13, *Figure* S3b). There was no enrichment of specific pathways amongst the DMRs associated with sarcopenia, but many of these lie within intergenic regions.

### Appendicular lean mass index, grip strength, and gait speed‐associated differentially methylated CpGs were enriched in pathways associated with calcium channel activity and cell adhesion

The PPI enrichment *P*‐value for the ALMi associated dmCpGs was *P* = 0.00345 (*Figure* S4a), although there were no significant GO terms associated with the whole network. Subdivision of the network showed enrichment for pathways involved in calcium channel activity and the sarcoplasmic reticulum (*Table* S14). For grip strength, the PPI network showed a significant enrichment for cell adhesion (FDR = 0.040), while for gait speed, there was enrichment of cadherin‐associated pathways (FDR ≤ 1.59 × 10^−11^). There was no enrichment of specific pathways amongst the DMRs associated with ALMi, grip strength, or gait speed.

### Sarcopenia‐associated differentially methylated CpGs were enriched in regions overlapping sites of H3K27me3 and EZH2 binding motifs

To gain further insights into the functional significance of the changes in DNA methylation, the dmCpGs were examined for enrichment within regions associated with specific histone modifications or transcription factor binding sites. There was a significant enrichment of sarcopenia‐associated dmCpGs overlapping polycomb repressed regions of the genome (FDR = 4.20 × 10^−4^) and Histone H3 lysine 27 trimethylation (H3K27me3) modifications (FDR = 4.41 × 10^−5^), with a significant under‐representation of dmCpGs overlapping regions of Histone H3 lysine 4 monomethylation (H3K4me1) modifications (FDR = 1.20 × 10^−3^) (*Figure* S5).

There was also enrichment for the Enhancer of Zeste Homologue 2 (EZH2) binding motif at the transcription start sites (TSS) and gene bodies of genes with a dmCpG associated with sarcopenia (TSS, FDR = 4.20 × 10^−4^; Body, FDR = 3.40 × 10^−4^), ALMi (TSS, FDR = 1.65 × 10^−3^; Body, FDR = 3.96 × 10^−6^), and grip strength (TSS, FDR = 2.98 × 10^−5^; Body, FDR = 5.93 × 10^−5^) (*Table* 4). The CCCTC‐binding factor (CTCF) binding motif was enriched at the TSS of genes with a dmCpG associated with grip strength (FDR = 0.0142) and in the gene bodies of genes associated with sarcopenia (FDR = 2.73 × 10^−3^), ALMi (FDR = 0.0185), gait speed (FDR = 4.88 × 10^−3^), and grip strength (FDR = 1.23 × 10^−5^).

**Table 4:** ENCODE ChIP‐Seq significance results for overlap with identified differentially methylated CpGs

	No. of genes associated with dmCpGs	Factor	No. of genes enriched	FDR
**TSS/5′End (1500 bp upstream, 500 bp downstream)**				
Sarcopenia	379	EZH2	37	4.20 × 10^−4^
ALMi	257	EZH2	26	1.65 × 10^−3^
Gait speed	206	—	—	—
Grip strength	214	EZH2	27	2.98 × 10^−5^
		CTCF	63	1.42 × 10^−2^
**Whole gene body**				
Sarcopenia	379	EZH2	30	3.40 × 10^−4^
		CTCF	147	2.73 × 10^−3^
ALMi	257	EZH2	27	3.96 × 10^−6^
		CTCF	98	1.85 × 10^−2^
Gait speed	206	CTCF	85	4.88 × 10^−3^
Grip strength	214	CTCF	99	1.23 × 10^−5^
		EZH2	21	5.93 × 10^−5^

ALMi, appendicular lean mass index; dmCpGs, differentially methylated CpGs; FDR, false discovery rate; TSS, transcription start sites.

### The EZH2 inhibitor GSK343 increases PAX7 expression, reduces myotube fusion index, and increases oxidative respiration in human primary muscle‐derived myoblasts

**Figure 3:**
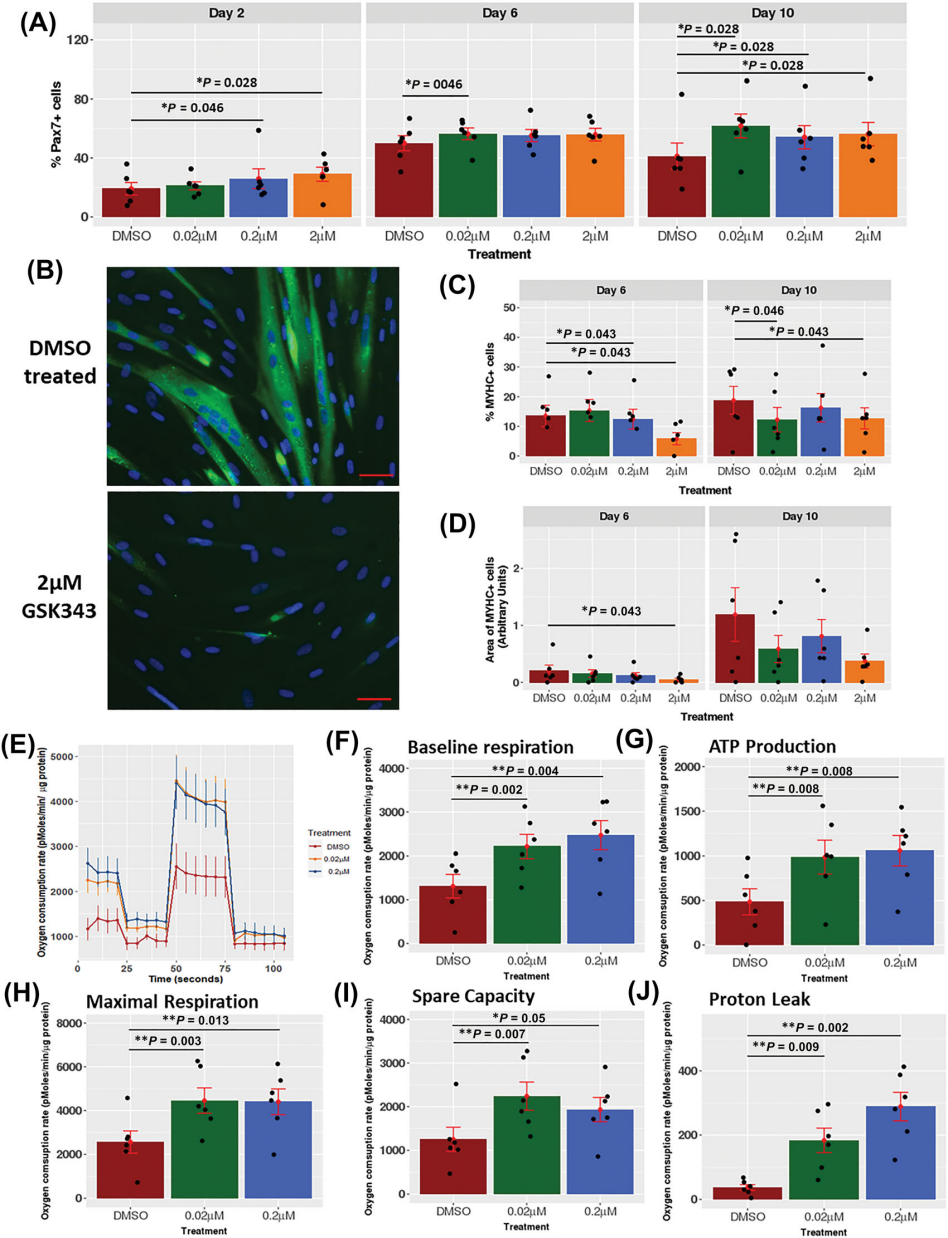
*(A)* The effect of pharmacological inhibition of EZH2 with GSK343 on human primary myoblast cells with respect to Pax7+ cell number at day 2, day 6, and day 10 of differentiation. *(B)* Representative stains of myosin heavy chain staining in the DMSO and GSK343‐treated cells. *(C)* Quantification of the fusion index after treatment with GSK343 at day 6 and day 10 of differentiation. Fusion index was measured as the percentage of MYHC+ cells with more than two nuclei. *(D)* Quantification of the area of the myosin heavy chain positive cells at day 6 and day 10 of differentiation. *(E)* Representative Seahorse trace of the mitochondrial stress test of GSK343‐treated cells after 10 days of differentiation. *(F–J)* The effect of GSK343 treatment at 200 and 20 nM on cells after 10 days of differentiation on baseline respiration *(F)*, ATP production *(G)*, maximal respiration *(H)*, spare capacity *(I)*, and proton leak *(J)*. Results shown are all *n* = 6, mean ± SEM. *P < 0.05, **P < 0.01.

As the sarcopenia‐associated dmCpGs were enriched amongst EZH2 target genes, as well as pathways including oxidative phosphorylation and myotube fusion, two pathways critical for myogenic differentiation, we investigated the role that EZH2 plays in the regulation of these pathways in primary human myoblasts *in vitro*. Human skeletal muscle‐derived myoblasts (*n* = 6) were treated with the specific EZH2 inhibitor, GSK343, and the expression of MYHC and PAX7 after the induction of differentiation, together with oxidative phosphorylation assessed. GSK343 led to an increase in PAX7 positive cells at D2, D6, and D10 (D2: 200 nM *P* = 0.046, 2 μM *P* = 0.028; D6: 20 nM *P* = 0.046; D10: 20 nM *P* = 0.028, 200 nM *P* = 0.028, 2 μM *P* = 0.028; *Figure* 3A). Concomitant with the increase in PAX7 staining, treatment of myoblasts with GSK343 resulted in a decrease in fusion index at D6 (*Figure* 3B and 3C; 200 nM *P* = 0.043, 2 μM *P* = 0.043) and at D10 (*Figure* 3B and 3C; 20 nM *P* = 0.046, 2 μM *P* = 0.043) compared with vehicle control‐treated cells. The reduction in fusion index was accompanied by a decrease in myotube area on D6 (*Figure* 3D; 2 μM *P* = 0.043), but no change in the intensity of MYHC staining at any time point.

The effect of GSK343 treatment on oxidative phosphorylation was assessed by measuring oxygen consumption rate (OCR) assessed at D10 of differentiation using the Seahorse XF96 analyser, as an indicator of oxidative phosphorylation (*Figure* 3E). Treatment with the GSK343 resulted in an increase in baseline respiration (*Figure* 3F; 20 nM *P* = 0.002, 200 nM *P* = 0.004), ATP production (*Figure* 3G; 20 nM *P* = 0.008, 200 nM *P* = 0.008), maximal respiration (*Figure* 3H; 20 nM *P* = 0.003, 200 nM *P* = 0.013), spare capacity (*Figure* 3I; 20 nM *P* = 0.007, 200 nM *P* = 0.050), and proton leak (*Figure* 3J; 20 nM *P* = 0.009, 200 nM *P* = 0.050).

### GSK343 alters DNA methylation at oxidative phosphorylation and myogenesis genes

To investigate whether treatment of the myoblasts with GSK343 affected the methylation of genes involved in oxidative phosphorylation and myogenesis, DNA methylation was assessed in vehicle control (DMSO) and GSK343 (200 nM) treated myoblasts (*n* = 5), on D10 after the induction of differentiation, when a reduction in myotube fusion and changes in oxidative respiration were observed. Treatment with GSK343 significantly changed the methylation at 112 CpGs out of 2637 probes in the KEGG oxidative phosphorylation geneset (*P* < 0.05; *Table* S15), with 78.6% of the CpGs hypomethylated following GSK343 addition. The top dmCpG was cg11189039 located within the NADH dehydrogenase [ubiquinone] 1 alpha subcomplex subunit 6 (*NDUFA6*) gene (*P* = 0.0012), a subunit of Complex 1 in the respiratory chain. Treatment with GSK343 also led to differential methylation at 352 CpGs out of the 9136 probes in the myogenesis geneset (*P* < 0.05); 78.7% of the CpGs were hypomethylated following EZH2 inhibition. Comparison of the dmCpGs (*P* < 0.05) changed in response to GSK343 treatment in the cultured myoblasts with those associated with sarcopenia in muscle tissue (FDR < 0.05) revealed that only seven of sarcopenia‐associated dmCpGs were also changed by GSK343 treatment in the cultured myoblasts; in all cases, these CpG sites were hypomethylated by GSK343 treatment.

## Discussion

Here, we report widespread changes in the muscle methylome associated with sarcopenia and individual measures of grip strength, lean mass, and gait speed, with significant overlap between the dmCpGs associated with sarcopenia and ALMi, sarcopenia and gait speed, and sarcopenia and grip strength. The dmCpGs associated with sarcopenia, and measures of muscle mass, strength, and function were enriched in genes involved in myotube fusion, oxidative phosphorylation, and voltage‐gated calcium channels. Moreover, examination of the chromatin landscape of the sarcopenia‐associated dmCpGs found that they were localized to EZH2 target genes and regions of H3K27 trimethylation. Furthermore, treatment of human primary myoblasts with a selective EZH2 inhibitor led to changes in oxidative phosphorylation and myotube fusion, together with altered methylation of genes involved in oxidative phosphorylation and myogenesis. Such data suggest that epigenetic changes may play an important role in muscle function and the aetiology of sarcopenia.

Significant changes in DNA methylation at both the single CpG and regional level were found to be associated with sarcopenia, many within intergenic enhancer regions.^31^ Of the sarcopenia‐associated dmCpGs that were associated with a gene, cg22843429 was located within the *EMG1* gene, an N1‐specific pseudouridine methyltransferase involved in ribosome biogenesis, while cg10977501 was located within *MYOM2* (Myomesin 2), a major component of the myofibrillar M‐band of the sarcomere, suggesting that differential methylation at these sites may contribute to the impairments in muscle function during ageing. The most significant DMR associated with sarcopenia was located within the *MCCC1* gene, a mitochondrial enzyme, which catalyses the carboxylation of 3‐methylcrotonyl CoA to 3‐methylglutaconyl CoA, a critical step in leucine catabolism. Mutations within this gene result in MCCC1 deficiency, which leads to metabolic acidosis, low plasma glucose and carnitine levels, and loss of muscle tone.^32^ In this study, both differential methylation and expression of *MCCC1* were observed in sarcopenic muscle, potentially impairing the availability of acetyl CoA and muscle metabolism. Interestingly, there was minimal overlap between the dmCpGs identified in this study (sarcopenia 8.5%, ALMi 5.6%, gait speed 4.3%, grip strength 12.2%) and those reported to differ in muscle tissue from young versus old individuals in the meta‐analysis by Voisin *et al*.^15^ Consistent with this, sarcopenia and its component measures were not associated with accelerated epigenetic muscle ageing, suggesting that sarcopenia is not an accentuated form of muscle ageing but rather involves distinct epigenetic changes.

Comparison of the muscle transcriptome from older individuals with sarcopenia and healthy aged‐matched controls found that the major transcriptional signal associated with sarcopenia was mitochondrial dysfunction.^17^ We found that oxidative phosphorylation was also one of the pathways enriched amongst the sarcopenia‐associated dmCpGs, suggesting that altered DNA methylation may mediate or consolidate the transcriptional changes observed in sarcopenic muscle. However, in the 34 samples with both RNAseq and methylation data, only 9.7% of the dmCpGs and 2.2% of the CpGs within the DMRs showed a significant correlation between DNA methylation and gene expression. This low level of correlation between DNA methylation and gene expression suggests that differential methylation of a number of CpGs may be required before there is a change in gene expression.

The sarcopenia‐associated, grip strength‐associated, and gait speed‐associated dmCpGs were also enriched in genes involved in homophilic cell–cell adhesion. Homophilic cell–cell adhesion plays a critical role in myotube fusion.^33^ Differential methylation of genes involved in myotube fusion in muscle biopsies from individuals with sarcopenic may impair the satellite cell (SC) repair mechanisms by inhibiting the fusion of the newly formed myotubes with existing myofibres. There was also enrichment amongst the sarcopenia and ALMi‐associated dmCpGs of genes involved in voltage‐gated Ca^2+^ channels, which are essential for the depolarization‐induced contraction of muscle cells, with impairments in calcium signalling and depolarization being linked to muscle atrophy.^34^ In muscle ageing studies, enrichment of homophilic cell adhesion^12^, ^13^ and calcium signalling^15^ have also been reported amongst ageing‐associated dmCpGs, suggesting that although the dmCpGs associated with ageing and sarcopenia differ, there is overlap in the pathways affected.

The sarcopenia‐associated dmCpGs were enriched within EZH2 target genes and sites of H3K27 trimethylation. Hypermethylation of EZH2 target genes have also been reported across a number of tissue types during ageing.^15^, ^35^ Although in sarcopenia, there was both hypermethylation and hypomethylation of the dmCpGs enriched in EZH2 target genes, suggesting epigenetic dysregulation of the EZH2 pathway in sarcopenia, rather than over‐activity or under‐activity of the EZH2 pathway. To investigate the role of EZH2 in myogenic cells, human primary myoblasts were treated with GSK343, an EZH2 inhibitor. GSK343 treatment led to an increase in ATP production, as well as increases in the maximal and basal rates of respiration, suggesting that inhibition of EZH2 signalling, either directly or indirectly, alters muscle cell metabolism. As differentiation of myoblasts into mature myotubes requires a metabolic switch from a highly glycolytic state to rely on oxidative phosphorylation (OXPHOS),^36^ increased oxidative phosphorylation might be expected to lead to an increase in differentiation and fusion index. However, treatment with GSK343 led to a decrease in myotube fusion with a concomitant increase in PAX7 expression. Studies have shown that while EZH2 plays a key role in the homeostasis of the adult muscle SC pool, upon SC differentiation, EZH2 re‐locates from myogenic genes, to repress self‐renewal genes such as PAX7 in differentiated myotubes.^37^ Inhibition of EZH2 may result in the de‐repression of self‐renewal genes like *PAX7* inhibiting muscle differentiation and myotube fusion in the differentiating myoblasts, despite the increased energetic capacity of the myoblasts.

The functional changes induced by GSK343 were accompanied by changes in the DNA methylation status of genes involved in OXPHOS and myogenesis, suggesting that these pathways are key targets of EZH2 regulation in primary myoblasts. Recent studies have demonstrated that EZH2 can recruit DNA methyltransferases to target genes, with EZH2 knockdown decreasing both H3K27 trimethylation and DNA methylation at these sites.^38^ Consistent with these previous findings, GSK343 treatment of the myoblasts altered DNA methylation, with the majority of CpG sites being hypomethylated. Comparison of the sarcopenia‐associated dmCpGs with those altered by GSK343 treatment showed that the methylation of only seven sarcopenia‐associated dmCpGs were affected by GSK343 treatment. The epigenetic landscape however of muscle tissue compared with the cultured myoblasts is likely to differ markedly; GSK343 treatment of myoblasts did however lead to an increase in oxidative respiration, suggesting that it may be possible to reset the epigenetic regulation of oxidative phosphorylation to improve muscle bioenergetics though manipulation of these pathways.

There were limitations to this study. Firstly, we only measured DNA methylation in male participants of the HSS and HSSe, and further studies are required to determine whether similar epigenetic changes occur in females. Secondly, we found that the majority of methylation changes were located within intergenic regions, so further work will be required to determine whether these changes contribute to changes in transcription. Thirdly, although GSK343 is a highly selective inhibitor of EZH2,^30^ confirmation that GSK343 treatment inhibited EZH2 activity is required together with the effect of EZH2 on the expression of myogenic and metabolic genes to precisely map the role that EZH2 plays in myotube fusion and mitochondrial bioenergetics. Finally, we tested the role of EZH2 in a myogenic enriched population of cells isolated from muscle biopsies; it would also be highly informative to assess the role of EZH2 in all muscle‐derived cells, which might be more representative of the aged muscle niche, as well as from a larger number of individuals to specifically test whether GSK3343 can alleviate the oxidative phosphorylation impairment observed in sarcopenic muscle.

## Conclusions

These findings show widespread changes in the muscle methylome associated with sarcopenia and individual measures of grip strength, lean mass, and gait speed. Furthermore, we show that treatment of human primary myoblasts with an inhibitor of EZH2 led to changes in oxidative phosphorylation and myotube fusion, together with altered DNA methylation of genes involved in oxidative phosphorylation and myogenesis. These findings support the premise that epigenetic processes play a central role in muscle function and suggest that it may be possible to ameliorate impairments in ATP production observed in muscle cells from individuals with sarcopenia though epigenetic manipulation of these pathways.

## Conflict of interest

A. Taddei and P. Richardson are full‐time employees of BenevolentAI Ltd. J.D. Holbrook is a full‐time employee of Cambridge Epigenetix. E. Migliavacca and J.N. Feige are full‐time employees of the Société des Produits Nestlé (SPN). K.M. Godfrey and H.P. Patel have received reimbursement for speaking at conferences sponsored by companies selling nutritional products. C. Cooper has received consultancy fees and honoraria from Amgen, Danone, Eli Lilly, GlaxoSmithKline, Medtronic, Merck, Nestlé, Novartis, Pfizer, Roche, Servier, Shire, Takeda, and UCB. E. Antoun, E.S. Garratt, M.A. Burton, S.J. Barton, P. Titcombe, K.M. Godfrey, and K.A. Lillycrop are part of academic research programmes that have received research funding from Abbott Nutrition, Nestec, and Danone. L.D. Westbury, A. Baczynska, H.E. Sydall, E. Dennison, R. Dodds, H.C. Roberts, A.A. Sayer, and S. Shaw declare that they have no conflicts of interest.

## Funding

This work was supported by collaborative projects with BenevolentAI Ltd and the Nestlé Institute of Health Sciences (NIHS) and grant funding from the Medical Research Council (MC_U47585827, MC_ST_U2055), Arthritis Research UK, National Osteoporosis Society, International Osteoporosis Foundation, Cohen Trust, NIHR Southampton Biomedical Research Centre, University of Southampton and University Hospital Southampton NHS Foundation Trust, NIHR Musculoskeletal Biomedical Research Unit, and University of Oxford. K.M.G. is supported by the UK Medical Research Council (MC_UU_20/4), the US National Institute On Aging of the National Institutes of Health (award number U24AG047867), the UK Economic and Social Research Council and the Biotechnology and Biological Sciences Research Council (award number ES/M0099X/), the National Institute for Health Research (as an NIHR Senior Investigator (NF‐SI‐055‐0042), and through the NIHR Southampton Biomedical Research Centre, and the European Union's Erasmus + Capacity‐Building ImpENSA Project. H.P.P. is supported by the National Institute for Health Research through the NIHR Southampton Biomedical Research Centre. This report is independent research, and the views expressed in this publication are those of the authors and not necessarily those of the NHS, the NIHR, or the Department of Health. The grant funders had no role in the design, collection, analysis, and interpretation of data, writing of the paper, or decision to submit for publication.

## Supporting information


**Data S1.** Supporting information

**Figure S1:** PCA of the top 50,000 most variable probes on the array to determine whether the samples group based on cohort as the DNA extraction method differed between the 2 cohorts. As there was no clear separation between the two, DNA extraction method was not accounted for in the analysis.
**Figure S2:** Correlation between epigenetic age as estimated by the muscle epigenetic age estimator MEAT, and chronological age.
**Figure S3:** (A) Protein–protein interaction (PPI) network generated from the genes associated with a CpG with an FDR < 0.2 with respect to sarcopenia. (B‐C) The PPI network was subdivided into smaller modules using the MCODE algorithm, of which 2 modules are shown.
**Figure S4:** PPI network of dmCpGs (A) associated with ALMI. Networks were further subdivided using the MCODE algorithm with two modules associated with the ALMI dmCpGs (B + C) shown.
**Figure S5:** (A) Enrichment of sarcopenia‐associated dmCpGs amongst 15 chromatin states as designated by the Epigenome Roadmap Project in male human skeletal muscle tissue samples. Odds ratio and significance calculated using the Fisher exact test. Heatmap shows the enrichment of the different histone modifications amongst the CpGs in the different chromatin states. (B) Enrichment of sarcopenia‐associated dmCpGs amongst six histone modifications as reported by ENCODE.

**Table S1**: List of Pyrosequencing primers
**Table S2:** List of Sarcopenia‐associated dmCpGs
**Table S3:** List of ALMi‐associated dmCpGs
**Table S4:** List of gait speed‐associated dmCpGs
**Table S5:** List of grip strength‐associated dmCpGs
**Table S6:** Overlap of the differentially methylated CpGs with respect to Sarcopenia, ALMi, Grip strength and Gait speed
**Table S7:** Association between sarcopenia, muscle mass, strength and function and muscle epigenetic age acceleration
**Table S8:** List of DMRs associated with sarcopenia
**Table S9:** List of DMRs associated with ALMi
**Table S10:** List of DMRs associated with grip strength
**Table S11:** List of DMRs associated with gait speed
**Table S12:** Significant GO terms associated with the Sarcopenia PPI network
**Table S13:** Top 10 GO terms associated with the top 2 sarcopenia‐associated clusters
**Table S14:** Top 10 GO terms associated with the top 2 clusters associated with the ALMI dmCpGs
**Table S15:** List of dmCpGs (*p* < 0.05) following GSK343 treatment after 10 days of differentiation of human primary myoblasts
