# Supplementary material for: Epigenome‐wide association study of sarcopenia: findings from the Hertfordshire Sarcopenia Study (HSS)
Source: J Cachexia Sarcopenia Muscle. 2021 Dec 4;13(1):240–53. doi: 10.1002/jcsm.12876 (PMC8818655; doi:10.1002/jcsm.12876)
Supplement: Supplementary file 1 — Data S1. Supporting information [file JCSM-13-240-s003.docx]

**Supplementary Methods**

**Study Design**

All participants were recruited from the Hertfordshire Cohort Study (HCS) ^1^, a retrospective cohort study based in the UK designed to investigate life course influences on muscle morphology, mass, strength and physical function in community-dwelling older people. DNA was analysed from 40 male participants from the first phase of the study, the Hertfordshire Sarcopenia Study (HSS), which has been described in detail previously ^2, 3^, and from the male participants of the second extension phase of the study (n=43) (2012-2015), which recruited a total of 168 male and female participants, herein termed HSSe ^4^. The 40 males from the HSS, and the 43 males from the HSSe were the only samples with sufficient DNA for both the genome wide methylation analysis and subsequent validation by pyrosequencing. All participants gave written informed consent and the study was approved by the Hertfordshire Research Ethics Committee (07/Q0204/68). Sarcopenia was defined according to the European Working Group on Sarcopenia in Older People (EWGSOP) ^5^ criteria, with the following thresholds: appendicular lean mass index (ALM/height^2^) ≤ 7.23 kg/m^2^ for men (≤ 5.67 kg/m^2^ for women); grip strength < 30 kg for men (< 20 kg for women); and walking speed ≤ 0.8 m/s. Participants were classed as healthy controls (normal ALMi, gait speed and grip strength) or as having sarcopenia (low ALMi and low gait speed and/or low grip strength).

**Procedures**

Height (cm) and weight (kg) were measured once. Body composition (total lean mass, appendicular lean mass [ALM], and fat mass) was assessed by dual-energy x-ray absorptiometry (DXA) (Hologic Discovery, software version 12.5) for all participants. Isometric grip strength (kg) was measured three times in each hand using a Jamar handheld hydraulic dynamometer (Promedics, UK) and the highest value of six measures was used for analysis^6^. Customary walking speed was measured over a 3m course. Percutaneous muscle biopsies of the *vastus lateralis* were conducted after an overnight fast under local anaesthetic using a Weil-Blakesley conchotome^7^. Approximately 30 mg of muscle tissue was used for cell culture, whilst 20mg was snap frozen in cooled isopentane and stored at -80°C until DNA extraction.

**DNA extraction**

Genomic DNA was extracted from muscle from HSS cohort participants using the QIAamp DNA mini kit (Qiagen) as per manufacturer’s instructions, and from HSSe cohort participants using the high salt method ^8^. Briefly, muscle tissue was homogenized in TNES (50mM Tris pH 7.5, 400mM NaCl, 100mM EDTA, 0.5% SDS) buffer containing proteinase K. 2.6M NaCl was added after complete digestion and DNA precipitated in 100% ethanol. DNA was RNase I treated and purified using a phenol-chloroform wash. DNA was finally precipitated using 3M sodium acetate and 100% ethanol and resuspended in DNase-free water. Quality and quantity of genomic DNA was checked on the NanoDrop ND-1000 (NanoDrop Technologies).

**RNA extraction**

RNA was extracted from frozen muscle tissue using the mirVana^TM^ miRNA Isolation Kit (Ambion, Life Technologies), following the protocol for total RNA extraction. RNA was quantified using Qubit 2.0 Fluorometer (Thermo Scientific), 260/280 and 260/230 ratios checked using a NanoDrop 1000 Spectrophotometer (Thermo Scientific) and run on the Agilent Bioanalyzer to confirm RNA integrity.

**Infinium HumanMethylation EPIC BeadChip array**

DNA methylation was investigated in muscle tissue samples using the Infinium Human MethylationEPIC BeadChip array. 750ng of genomic DNA was treated with Sodium bisulfite using Zymo EZ DNA Methylation-Gold kit (ZymoResearch, Irvine, California, USA) and processing of the Human MethylationEPIC (Illumina, Inc. CA, USA) platform was carried out by the Centre for Molecular Medicine and Therapeutics (CMMT) (<http://www.cmmt.ubc.ca)>.

**Infinium Human MethylationEPIC BeadChip array data processing**

Infinium 850K data was processed using the Bioconductor package minfi ^9^ in R (version 3.4.2). We applied beta-mixture quantile (BMIQ) normalization to remove array biases and correct for probe design. Methylation profiling was carried out in 88 muscle tissue samples, which included 5 technical replicates. CpGs known to cross-hybridise to other locations in the genome (n=14,759) or coinciding with SNPs (n=77,261) were removed from the analysis. Probes with a detection p-value > 0.01 (n=5,604) and beadcount < 3 (n=1,051) were removed from the dataset. 17,614 probes aligning to the sex chromosomes and 2,928 non-CpG probes were also removed from the dataset. One sample did not have any intensity results so was discarded. Five duplicate samples were included, for which the Euclidean distance was calculated, and hierarchical clustering, using complete linkage clustered the pairs together. Data was further assessed by visualization of methylation density plots and calculation of median absolute deviation (MAD) scores. Duplicates were removed after normalization but before inference (the duplicate with the lowest MAD score was removed). Six samples showed aberrant methylation densities and MAD scores lower than -5 were removed from subsequent analysis. This resulted in 77 samples which were taken forward for further analysis. As the DNA was extracted using different methods for HSS and HSSe, principal component analysis (PCA) was carried out to determine whether the samples clustered on the basis of the DNA extraction method (Supplementary Figure 1). There was no separation between the two cohorts; therefore, the analysis was not adjusted for DNA extraction method. The methylation data of the muscle tissue can be accessed on the gene expression omnibus (https://www.ncbi.nlm.nih.gov/geo/), under accession number GSE154980.

To assess the effect of GSK343 treatment on DNA methylation in cultured myoblasts, EPIC arrays were run on DNA samples extracted from myoblasts in the presence or absence of 20nM GSK343 for 10 days after the addition of differentiation media (n=6 per group) and processed as above. One sample with a MAD score < -5 was discarded. CpGs known to cross-hybridise to other locations in the genome (n=40,571) or coinciding with SNPs (n=30,820) were removed from the analysis. Probes with a detection p-value > 0.01 (n=809) and beadcount < 3 (n=7,206) were removed from the dataset. 17,614 probes aligning to the sex chromosomes and 2,928 non-CpG probes were also removed from the dataset. The methylation data of the GSK343 treated myoblasts can be accessed on the gene expression omnibus (https://www.ncbi.nlm.nih.gov/geo/), under accession number xxx.

**Infinium Human MethylationEPIC BeadChip array data analysis**

*Methylation profiling in muscle tissue samples:* ComBat was run in order to adjust the data for the batch effect arising due to the different chips the samples were run on. Robust regression models using limma ^10^ were run with methylation as the outcome variable. All models included the participant’s age as a covariate. Sex was not included in the models as all samples were from male participants. As DNA methylation is generally tissue specific and muscle tissue is not comprised of a homogenous cell population, surrogate variable analysis (SVA) was carried out to account for any variations in DNA methylation that may arise due to differences in cellular heterogeneity. The variables calculated by SVA were included in the model alongside age as covariates. The analysis was controlled for multiple testing with the Benjamini-Hochberg adjustment for false discovery rate.

*Methylation profiling of cultured myoblasts:* Paired t-test analysis was carried out on the 2637 probes that were associated with the 135 genes in the KEGG oxidative phosphorylation geneset (MSigDB) according to the Illumina annotation in the GSK treated and untreated cells. Paired t-test analysis was also carried out on the 9136 probes that were associated with the 200 genes in the Hallmark myogenesis geneset and 27 genes in the Reactome striated muscle contraction geneset (MSigDB) according to the Illumina annotation.

**Muscle Epigenetic Age Estimator (MEAT)**

Epigenetic age acceleration was calculated as the residuals of regressing the epigenetic age estimated by the muscle epigenetic age estimator (MEAT) ^14^ over chronological age using the MEAT package in R. A positive residual suggests faster aging, and a negative values suggests a slower aging.

**Network and Gene Ontology Enrichment**

Protein-protein interaction (PPI) networks were carried out using the Search Tool for the Retrieval of Interacting Genes/Proteins (STRING) using the genes associated with a dmCpG (FDR<0.2) and visualized in Cytoscape. The properties of the PPI network were calculated under default parameters and only connected nodes were retained for further analysis. Large networks were further segmented using the MCODE algorithm ^11^ in Cytoscape using default parameters, in order to conduct module analysis of the PPI network. Enriched gene ontology (GO) terms were determined using BiNGO ^12^ to examine overrepresented GO terms.

**RNA sequencing and analysis**

RNA sequencing was carried out as described in Migliavaca et al. ^13^. Briefly, 250 ng of total RNA was used as input for the TruSeq Stranded Total RNA HT kit with the Ribo-Zero Gold module (Illumina), libraries amplified using the KAPA HiFi HotStart ReadyMix (Kapa BioSystems), quantified with Picogreen (Life Technologies) and size pattern was controlled with the DNA High Sensitivity Reagent kit on a LabChip GX (Perkin Elmer). Libraries were pooled at an equimolar ratio and sequencing was performed for 2 x 101 cycles on a HiSeq 2500 (Illumina) with v3 chemistry. The generated data were demultiplexed using Casava. Reads were aligned to the human reference genome (GRCh38) using the TopHat aligner (version 2.0.14) and the number of reads mapped to a gene was quantified using HTSeq (version 0.6.1). Normalized counts were generated for each gene and used for downstream analysis. Lowly expressed genes (log(count)<5 in more than 20 samples) were removed from the dataset. The unprocessed transcriptomic data can be accessed on the gene expression omnibus (<https://www.ncbi.nlm.nih.gov/geo/>), under accession number GSE111006.

**Enrichment of dmCpGs among chromatin enhancer states and histone modifications**

We obtained the ChIP-seq peak regions in the broadPeak format for processed ENCODE ChIP-seq experiments in male human skeletal muscle tissue from the ENCODE data portal (https://www.encodeproject.org). The 15-state chromatin model for skeletal muscle tissue was obtained from the Epigenome Roadmap. We assessed the enrichment of dmCpGs among the chromatin states and histone modifications using the Fisher exact test, with all the CpGs on the array as a background. Transcription factor enrichment was carried out using the ENCODE ChIP-seq enrichment tool, using data from human skeletal muscle myoblasts and myotubes.

**Quantitative DNA Methylation analysis**

500ng of genomic DNA was bisulfite-converted using the EZ DNA Methylation Gold Kit (Zymo Research, Ivrine, CA), as per the manufacturer’s protocol. PCR primers specific for bisulfite-converted DNA were designed using the PyroMark Assay Design Software v2.0 (Qiagen). Primer sequences are shown in Supplementary Table 1. Quantitative DNA methylation analysis was carried out by pyrosequencing. Bisulfite-converted DNA was amplified (Qiagen HotStar Taq Polymerase) and PCR products were immobilised on streptavidin-sepharose beads (GE Healthcare), washed, denatured and released into annealing buffer containing the appropriate sequencing primer. Pyrosequencing was carried out on a PyroMark MD (Biotage) and methylation percentage calculated using the Pyro Q CpG software (Qiagen).

**Isolation of Myoblast cells from Muscle Biopsies**

All samples were processed within 1 hour of muscle biopsy. Briefly, biopsies were minced, then digested in 0.5 mg/ml collagenase (Sigma) in serum free Dulbecco's modified eagle medium (DMEM, Gibco) at 37°C for 20 min with agitation. Samples were then centrifuged and resuspended in phosphate-buffered saline (PBS) and subsequently filtered through a 100µM cell strainer (BD Falcon). Cells were centrifuged again and resuspended in proliferation medium (DMEM containing 20% fetal bovine serum (FBS), 10% Horse Serum (HS), 1% Chick embryo extract (CEE) (Sera Labs) and 1% Penicillin/Streptomycin (P/S)). Next, the cells were pre-plated onto 10 cm dishes and incubated at 37°C for 3 hours to permit any fibroblasts to attach. Cells and media were then transferred to 10cm matrigel coated dishes (BDBiosciences) and incubated for 48 hours. To enrich the myogenic population, cells were sorted using CD56 MicroBeads prior to experiments ^14^ (Miltenyi Biotech) according to the manufacturer’s instructions, and isolation of myogenic cells confirmed by immunocytochemistry with CD56 in myoblasts. All experiments were performed at passage 4.

**Differentiation of Myoblast Cells**

Cells were plated at 2,000 cells/cm^2^ (unless stated otherwise) and maintained in proliferation medium. When cells reached 90% confluence, they were washed in PBS and the media replaced with differentiation media (DMEM containing 2% horse serum and 1% P/S). This was termed differentiation day 0. Cells were subsequently maintained at 37˚C in a humid atmosphere containing 5% CO2 and media was refreshed every 48 hours until required for experiments.

**Epigenetic Inhibitor treatments**

Human primary myoblasts from six individuals from the HSSe were treated with GSK343 (MedChemExpress) at 20nM, 200nM and 2µM after the initiation of differentiation over a 10-day period. GSK343 is a selective **S-Adenosyl-L-methionine (**SAM)-competitive inhibitor of EZH2, with an IC50 of 200nM ^15^ which has been reported to show no significant inhibitory effects on other enzymes requiring SAM as cofactor, including DNA methyltransferase (DNMT) and Protein Arginine Methyltransferase 1 (PRMT1) ^15^. GSK343 was prepared in dimethyl sulfoxide (DMSO) and diluted to final working concentrations immediately prior to use. The final concentration of DMSO was 0.1%.

**Immunocytochemistry**

Myogenic purity of sorted cell cultures was measured using immunocytochemistry with CD56 as a marker. To assess myotube fusion index, cells were stained for myosin heavy chain (MYHC) expression at differentiation day 2, day 6 and day 10 after the addition of differentiation media. In addition, cells were also stained for the satellite cell self-renewal marker PAX7 at the same time points. Briefly, cells were fixed in 3.7% **paraformaldehyde (**PFA) for 10 minutes at room temperature. Following a PBS wash, cells were then permeabilised (0.3% Triton X-100, 1% bovine serum albumin (BSA) in PBS) for 7 min at room temperature. Cells were then washed in PBS-Tween and blocked (5% goat serum, 1% BSA in PBS) for 1hr at room temperature. Cells were washed and incubated with the appropriate primary antibody overnight at 4°C with gentle agitation; CD56 1:50 (anti-NCAM, Abcam), myosin heavy chain (MYHC 1:50, MF-20, DSHB), PAX7 1:20 (anti-Pax7, Abcam). Cells were washed and incubated with secondary antibodies; goat anti-rabbit IgG AlexaFluor 594 1:200 (Abcam), goat anti-mouse IgG AlexaFluor 488 1:200 (Abcam). To visualize nuclei, cells were stained with 4′,6-diamidino-2-phenylindole (DAPI) (1µg/ml). Cells were imaged using the Axio observer D1 microscope (Zeiss). For immunocytochemistry, a 20X objective was used. Five images of randomly selected non-overlapping fields were captured for each well. Cells on unadjusted images were counted using ImageJ. To calculate the fusion index, the number of nuclei incorporated into the myotubes (containing 2+ nuclei) was counted and the ratio of this number to the total number of nuclei was determined. ImageJ was also used to measure the area and intensity of individual MYHC-positive myotubes in each non-overlapping field, and the mean area and intensity of each well was calculated.

**Metabolic Flux Assay**

Mitochondrial bioenergetics were measured in attached cells using the Agilent Seahorse XF96 Mito Stress Test. Briefly, primary myoblasts were plated into XF96 culture plates in proliferation media. At 80% confluence, proliferation media was substituted for low-serum media (DMEM + 2% horse serum) to initiate differentiation. Experiments were carried out at day 10 of differentiation. Cells were washed in pre-warmed running media (DMEM + 1% P/S + 2mM L-glutamine, pH7.4) and incubated in running media for 1hr in a non-CO2 incubator. For the Mito Stress test, pre-warmed oligomycin (ATP synthase inhibitor), Carbonyl cyanide-4-phenylhydrazone (FCCP, an uncoupling agent) and rotenone (electron transport chain inhibitor) & antimycin A were loaded into injector ports A, B and C of the sensor cartridge, respectively. After calibration by the XF96 analyzer, cells were loaded and the Cell Mito stress test assay performed. Oxygen consumption rate (OCR) were detected under basal conditions followed by the sequential injection of compounds. After completion, cells were lysed and a protein assay carried out to allow for normalization. Each data point represents the mean +/- SD of six replicates for each condition.

**Statistical analysis**

All statistical analysis was carried out in R (version 3.4.2). Demographic characteristics were compared between controls and those with sarcopenia using Mann–Whitney U tests. The hypergeometric distribution probability test was used to test the significance of the overlap between dmCpGs associated with different measures of muscle mass/function. Fisher’s exact test was used to test the enrichment of dmCpGs among the different histone modifications, chromatin enhancer states and genomic regions relative to CpG islands. Linear models were fitted to the pyrosequencing data including age as a covariate. Correlation analysis of the methylation and gene expression data was carried out using Spearman correlation. Statistical analysis of the GSK343 treated cell cultures was carried out using the Wilcoxon signed-rank test for the immunocytochemistry and paired t-test for the metabolic flux assays.

**References**

1. Syddall HE, Aihie Sayer A, Dennison EM, Martin HJ, Barker DJ, Cooper C. Cohort profile: The hertfordshire cohort study. *Int J Epidemiol*. 2005;34:1234-1242

2. Patel HP, Syddall HE, Martin HJ, Stewart CE, Cooper C, Sayer AA. Hertfordshire sarcopenia study: Design and methods. *BMC Geriatr*. 2010;10:43

3. Syddall HE, Simmonds SJ, Carter SA, Robinson SM, Dennison EM, Cooper C, et al. The hertfordshire cohort study: An overview. *F1000Res*. 2019;8:82

4. Westbury LD, Dodds RM, Syddall HE, Baczynska AM, Shaw SC, Dennison EM, et al. Associations between objectively measured physical activity, body composition and sarcopenia: Findings from the hertfordshire sarcopenia study (hss). *Calcif Tissue Int*. 2018;103:237-245

5. Cruz-Jentoft AJ, Baeyens JP, Bauer JM, Boirie Y, Cederholm T, Landi F, et al. Sarcopenia: European consensus on definition and diagnosis: Report of the european working group on sarcopenia in older people. *Age Ageing*. 2010;39:412-423

6. Roberts HC, Denison HJ, Martin HJ, Patel HP, Syddall H, Cooper C, et al. A review of the measurement of grip strength in clinical and epidemiological studies: Towards a standardised approach. *Age Ageing*. 2011;40:423-429

7. Patel H, Syddall HE, Martin HJ, Cooper C, Stewart C, Sayer AA. The feasibility and acceptability of muscle biopsy in epidemiological studies: Findings from the hertfordshire sarcopenia study (hss). *J Nutr Health Aging*. 2011;15:10-15

8. Aljanabi SM, Martinez I. Universal and rapid salt-extraction of high quality genomic DNA for pcr-based techniques. *Nucleic Acids Res*. 1997;25:4692-4693

9. Aryee MJ, Jaffe AE, Corrada-Bravo H, Ladd-Acosta C, Feinberg AP, Hansen KD, et al. Minfi: A flexible and comprehensive bioconductor package for the analysis of infinium DNA methylation microarrays. *Bioinformatics*. 2014;30:1363-1369

10. Ritchie ME, Phipson B, Wu D, Hu Y, Law CW, Shi W, et al. Limma powers differential expression analyses for rna-sequencing and microarray studies. *Nucleic Acids Res*. 2015;43:e47

11. Bader GD, Hogue CW. An automated method for finding molecular complexes in large protein interaction networks. *BMC Bioinformatics*. 2003;4:2

12. Maere S, Heymans K, Kuiper M. Bingo: A cytoscape plugin to assess overrepresentation of gene ontology categories in biological networks. *Bioinformatics*. 2005;21:3448-3449

13. Migliavacca E, Tay SKH, Patel HP, Sonntag T, Civiletto G, McFarlane C, et al. Mitochondrial oxidative capacity and nad(+) biosynthesis are reduced in human sarcopenia across ethnicities. *Nat Commun*. 2019;10:5808

14. Agley CC, Rowlerson AM, Velloso CP, Lazarus NL, Harridge SD. Isolation and quantitative immunocytochemical characterization of primary myogenic cells and fibroblasts from human skeletal muscle. *J Vis Exp*. 2015:52049

15. Verma SK, Tian X, LaFrance LV, Duquenne C, Suarez DP, Newlander KA, et al. Identification of potent, selective, cell-active inhibitors of the histone lysine methyltransferase ezh2. *ACS Med Chem Lett*. 2012;3:1091-1096
