# Supplementary material for: Epigenome‐wide association study of sarcopenia: findings from the Hertfordshire Sarcopenia Study (HSS)
Source: J Cachexia Sarcopenia Muscle. 2021 Dec 4;13(1):240–53. doi: 10.1002/jcsm.12876 (PMC8818655; doi:10.1002/jcsm.12876)
Supplement: Supplementary file 2 — Figure S1: PCA of the top 50,000 most variable probes on the array to determine whether the samples group based on cohort as the DNA extraction method differed between the 2 cohorts. As there was no clear separation between the two, DNA extraction method was not accounted for in the analysis. Figure S2: Correlation between epigenetic age as estimated by the muscle epigenetic age estimator MEAT, and chronological age. Figure S3: (A) Protein–protein interaction (PPI) network generated from the genes associated with a CpG with an FDR < 0.2 with respect to sarcopenia. (B‐C) The PPI network was subdivided into smaller modules using the MCODE algorithm, of which 2 modules are shown. Figure S4: PPI network of dmCpGs (A) associated with ALMI. Networks were further subdivided using the MCODE algorithm with two modules associated with the ALMI dmCpGs (B + C) shown. Figure S5: (A) Enrichment of sarcopenia‐associated dmCpGs amongst 15 chromatin states as designated by the Epigenome Roadmap Project in male human skeletal muscle tissue samples. Odds ratio and significance calculated using the Fisher exact test. Heatmap shows the enrichment of the different histone modifications amongst the CpGs in the different chromatin states. (B) Enrichment of sarcopenia‐associated dmCpGs amongst six histone modifications as reported by ENCODE. [file JCSM-13-240-s002.docx]

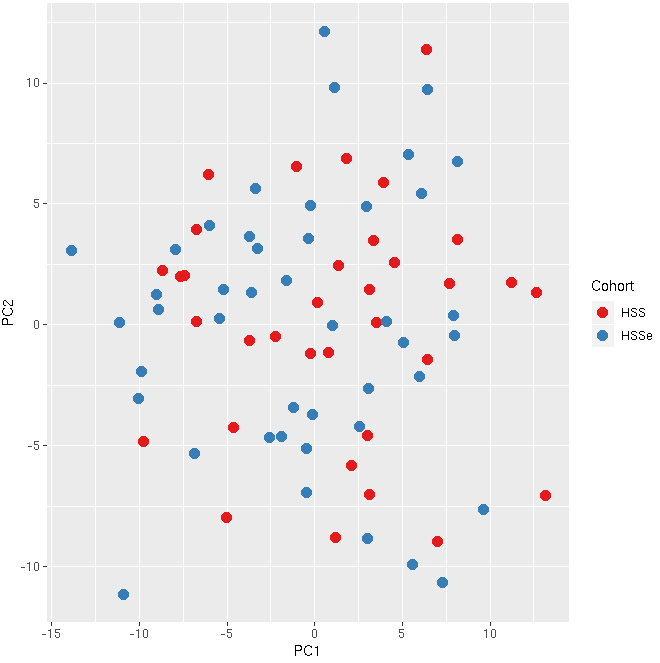
Supplementary figure 1: PCA of the top 50,000 most variable probes on the array to determine whether the samples group based on cohort as the DNA extraction method differed between the 2 cohorts. As there was no clear separation between the two, DNA extraction method was not accounted for in the analysis.


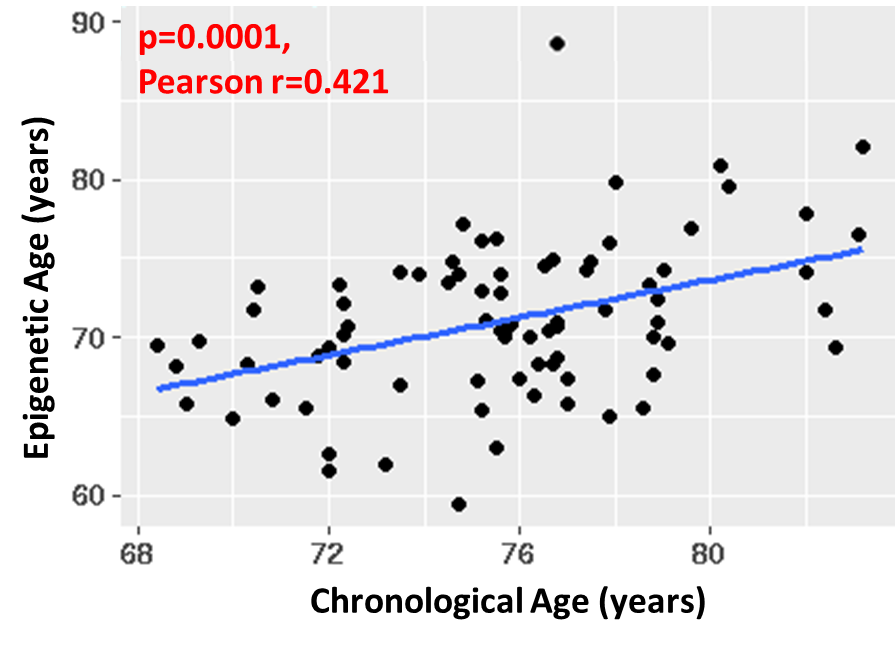
Supplementary Figure 2: Correlation between epigenetic age as estimated by the muscle epigenetic age estimator MEAT, and chronological age.


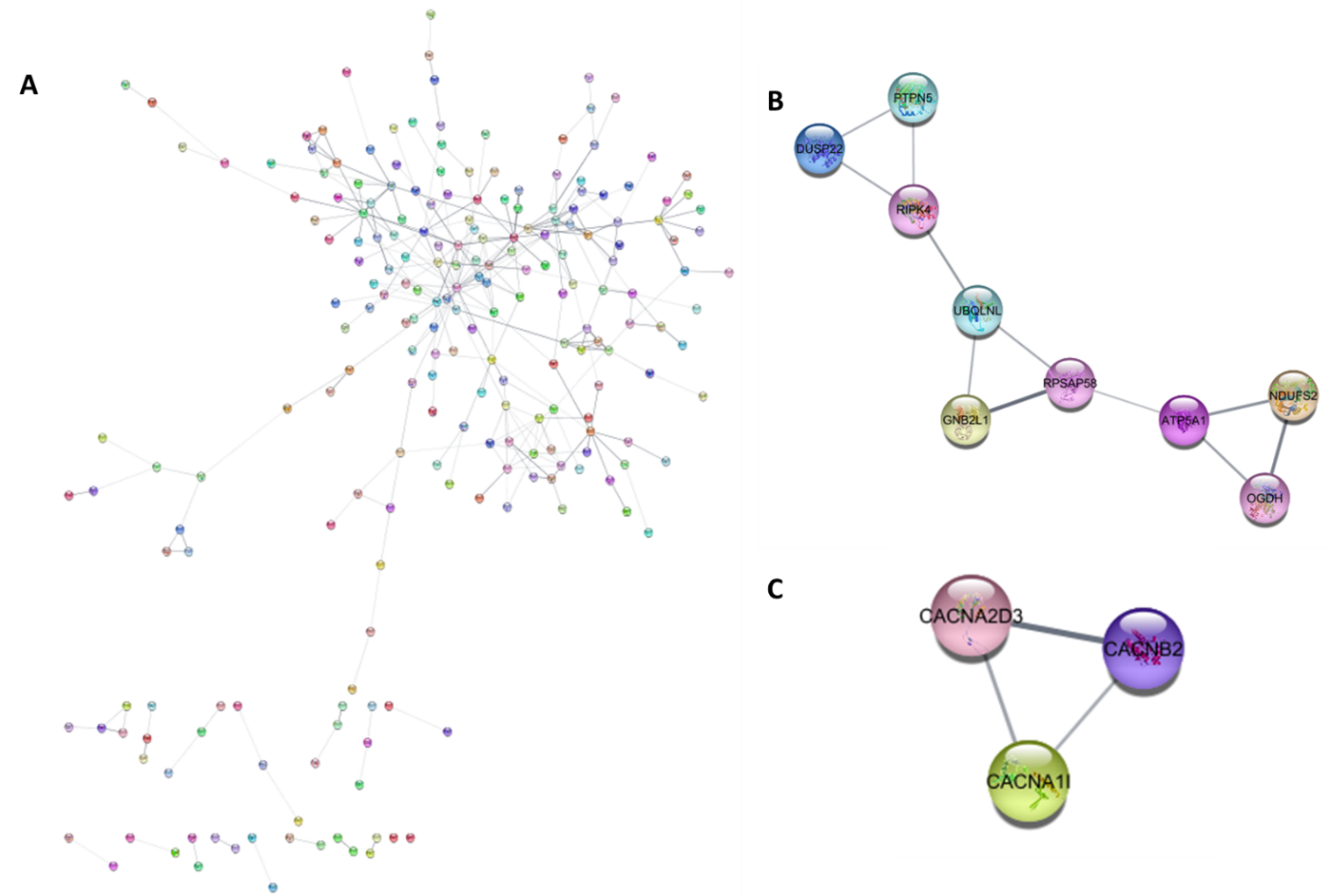


Supplementary Figure 3: (A) Protein-protein interaction (PPI) network generated from the genes associated with a CpG with an FDR<0.2 with respect to sarcopenia. (B-C) The PPI network was subdivided into smaller modules using the MCODE algorithm, of which 2 modules are shown.


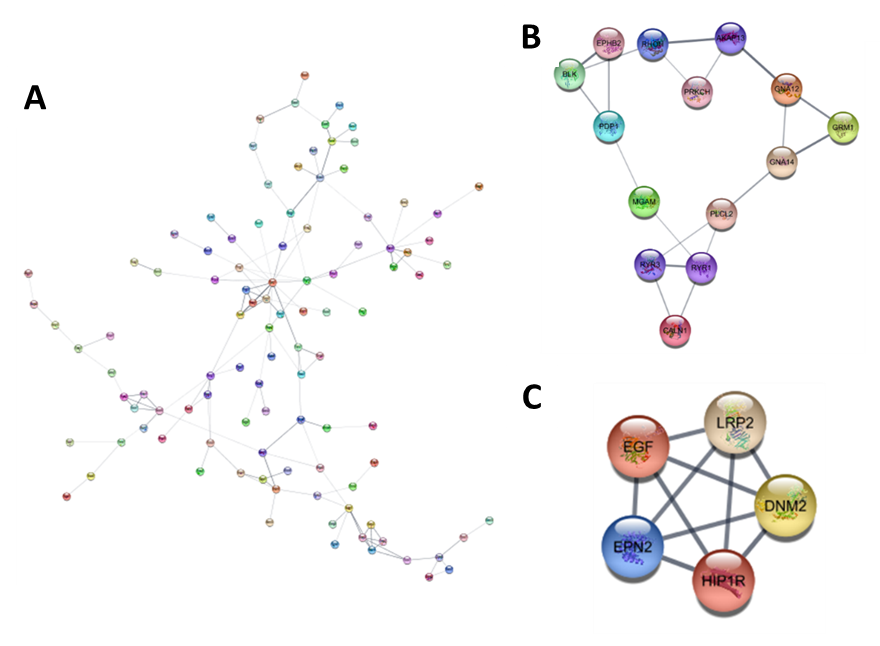


Supplementary Figure 4: PPI network of dmCpGs (A) associated with ALMI. Networks were further subdivided using the MCODE algorithm with two modules associated with the ALMI dmCpGs (B+C) shown.


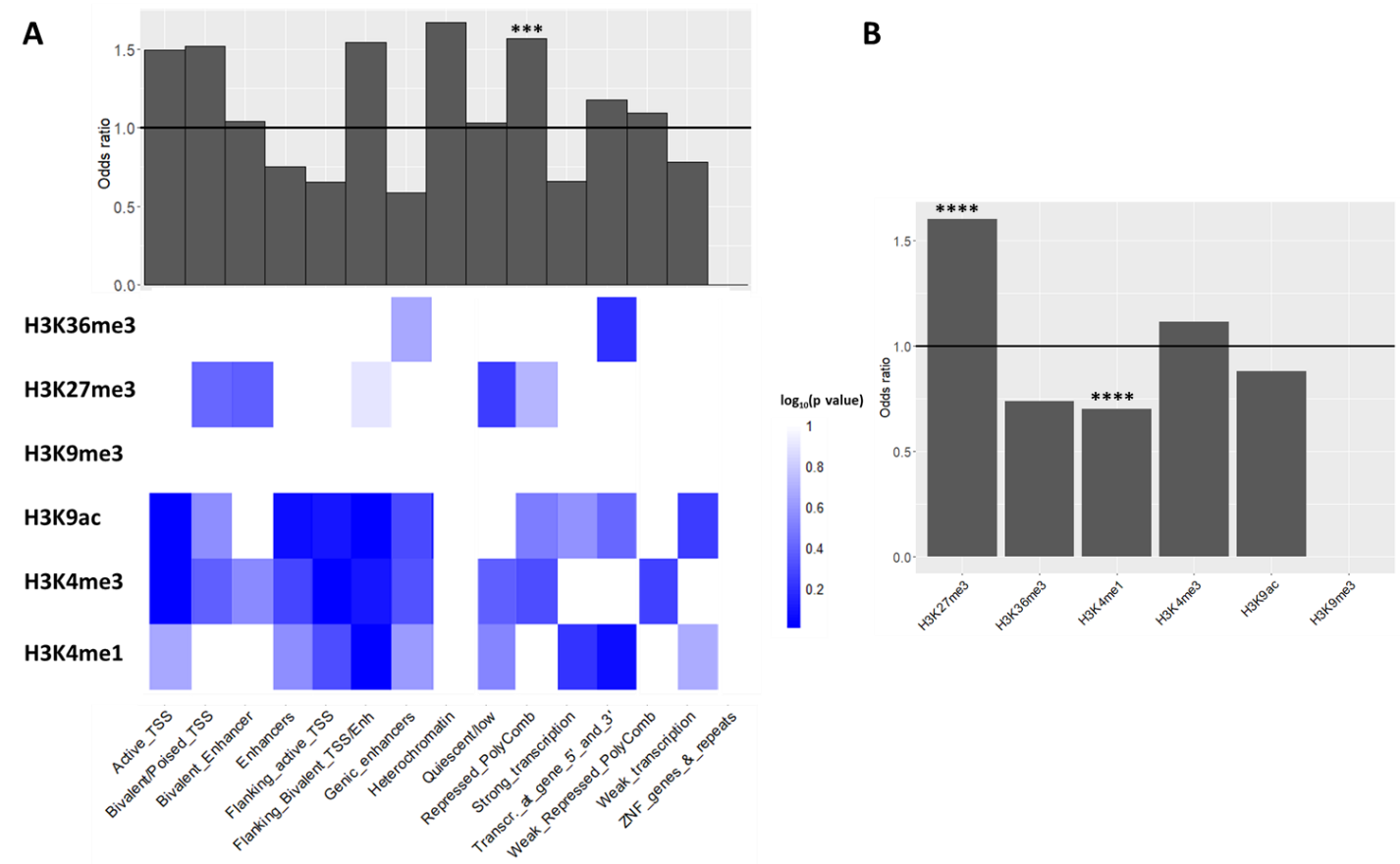


Supplementary Figure 5: (A) Enrichment of sarcopenia-associated dmCpGs amongst 15 chromatin states as designated by the Epigenome Roadmap Project in male human skeletal muscle tissue samples. Odds ratio and significance calculated using the Fisher exact test. Heatmap shows the enrichment of the different histone modifications amongst the CpGs in the different chromatin states. (B) Enrichment of sarcopenia-associated dmCpGs amongst six histone modifications as reported by ENCODE.
